# Upregulation of Apoptotic Protein and Migration-Inhibitory Effects of Gallic Acid and Methyl Gallate Combined with Cisplatin in HeLa Cervical Cancer Cells

**DOI:** 10.21315/tlsr2026.37.1.6

**Published:** 2026-03-31

**Authors:** Hasmah Abdullah, Norlida Mamat, Syahirah Sazeli, Hermizi Hapidin, Sarina Sulong, Illyana Ismail, Deena Elsori

**Affiliations:** 1 School of Health Sciences, Universiti Sains Malaysia Health Campus, 16150 Kubang Kerian, Kelantan, Malaysia; 2 School of Biomedicine, Faculty of Health Sciences, Universiti Sultan Zainal Abidin, Gong Badak Campus, 21300 Kuala Nerus, Terengganu, Malaysia; 3 Human Genome Centre, School of Medical Sciences, Universiti Sains Malaysia Health Campus, 16150 Kubang Kerian, Kelantan, Malaysia; 4 Academic and Vocational Affairs Section, Rabdan Academy, 114646 Abu Dhabi, United Arab Emirates

**Keywords:** Gallic Acid, Methyl Gallate, Cisplatin, Apoptotic Proteins, HeLa Cells, Asid Galik, Metil Galat, Cisplatin, Protein Apoptosis, Sel HeLa

## Abstract

The study investigated the effects of gallic acid (GA) and methyl gallate (MG), in combination with cisplatin (CIS), on apoptotic protein expression, antioxidant gene expression and cell migration in HeLa cervical cancer cells. HeLa cells were treated with the combinations CIS–GA and CIS–MG prior to further analysis. The expression of apoptotic proteins (Bax, Bcl-2, caspase-3, caspase-9 and p53) was determined via western blot analysis. Antioxidant gene expression in treated HeLa cells was then assessed using reverse transcription quantitative polymerase chain reaction (RT-qPCR). The migration-inhibitory effect of the compound combinations was assessed using a scratch wound-healing assay. Treatment with CIS–GA significantly upregulated the expression of p53, Bax, caspase-3 and caspase-9, and downregulated the expression of Bcl-2. Results from RT-qPCR revealed downregulation of human catalase (*hCAT*) in the CIS–GA group and no changes in superoxide dismutase 1 (*SOD1*) expression in either the CIS–GA or CIS–MG groups. Moreover, lower wound closure percentages were observed after 24-hour incubation in both treated groups, indicating inhibited cell migration. These findings suggest that GA and MG combined with CIS upregulate apoptotic proteins by downregulating antioxidant gene (*hCAT*).

## Abstrak

Kajian ini menyiasat kesan asid galik (GA) dan metil galat (MG) yang digabungkan dengan cisplatin (CIS) ke atas ekspresi protein apoptosis, ekspresi gen antioksidan dan migrasi sel dalam sel kanser serviks HeLa. Sel HeLa dirawat dengan gabungan CIS–GA dan CIS–MG sebelum dianalisis lanjut. Ekspresi protein apoptosis (Bax, Bcl-2, caspase-3, caspase-9 dan p53) ditentukan melalui analisis western blot. Ekspresi gen antioksidan dalam sel HeLa yang dirawat kemudiannya dinilai menggunakan kaedah reaksi rantai polimerase kuantitatif transkripsi songsang (RT-qPCR). Kesan penghambatan migrasi sel oleh gabungan sebatian ini dinilai menggunakan ujian penyembuhan luka calar. Rawatan CIS–GA secara signifikan meningkatkan ekspresi p53, Bax, caspase-3 dan caspase-9, serta menurunkan ekspresi Bcl-2. Keputusan RT-qPCR menunjukkan penurunan ekspresi gen katalase manusia (hCAT) dalam kumpulan CIS–GA dan tiada perubahan dalam ekspresi gen superoksida dismutase 1 (SOD1) bagi kumpulan CIS–GA dan CIS–MG. Selain itu, peratusan penutupan luka yang lebih rendah diperhatikan selepas inkubasi selama 24 jam dalam kedua-dua kumpulan yang dirawat, menunjukkan migrasi sel terhalang. Penemuan ini mencadangkan bahawa GA dan MG yang digabungkan dengan CIS meningkatkan protein apoptosis melalui penurunan ekspresi gen antioksidan (hCAT).

**HIGHLIGHTS**

- Combination treatments (CIS–GA and CIS–MG) induced apoptosis in HeLa cells by upregulating p53, Bax, caspase-3 and caspase-9, while downregulating Bcl-2.
- CIS–GA treatment specifically downregulated human catalase (*hCAT*) expression, with no significant changes observed in *SOD1*.
- Both CIS–GA and CIS–MG treatments reduced wound closure in the scratch assay, indicating inhibition of HeLa cell migration.

## INTRODUCTION

Cervical cancer is the fourth most frequent cancer and the fourth most common cause of death among women globally, accounting for an estimated 660,000 (6.8%) new cases and 350,000 (8.1%) deaths in 2022 (Bray *et al*. 2024). Cancer therapies typically involve chemotherapy, surgery, immunotherapy and radiotherapy. The use of chemotherapy to inhibit the proliferation of cancer cells remains a mainstay of treatment. Nevertheless, with continuous administration of chemotherapeutic drugs, cancer cells appear to become progressively resistant, leading to a gradual reduction in the potency of these agents (Marchetti *et al*. 2021). In addition, the major side effects of chemotherapy remain a concern that must be urgently addressed (Kim *et al*. 2021).

Chemotherapeutic drugs are frequently administered in combination rather than as single agents, since different drugs kill cancer cells through different mechanisms (National Cancer Institute 2021). Cisplatin (CIS), a platinum-based drug, is one of the most used active anticancer agents in treating cancers, either alone or in combination with other chemotherapeutic agents such as carboplatin and oxaliplatin (Dasari & Tchounwou 2014). However, despite its clinical efficacy, the use of CIS is often limited by chemoresistance and numerous side effects—including nephropathy, hearing loss, allergic reactions, decreased immune function, gastrointestinal problems and haemorrhage—prompting interest in alternative or complementary strategies.

Therefore, researchers have increasingly focused on natural products because of their effectiveness in combating various diseases and their relatively fewer side effects. The use of chemotherapeutic drugs in combination with natural substances is considered a promising cancer therapeutic approach (Kojima-Yuasa *et al*. 2015). It has been suggested that combining chemotherapeutic drugs with natural compounds may increase anticancer activity through synergistic interactions and help compensate for adverse effects (Sarkar & Li 2006). Numerous studies have reported the promising effects of combining CIS with several natural products on cervical cancer cells. For example, caffeine combined with CIS enhanced the cytotoxic effect of CIS on HeLa cervical cancer cells by lowering the IC_50_ value from 8.93 μM to 2.75 μM (Aliwaini *et al*. 2017). The combination of CIS and caffeic acid showed synergistic interaction on human cervical cancer cells by inducing apoptosis (Koraneekit *et al*. 2018). Synergistic cytotoxicity effects on HeLa cells were also observed after treatment with a combination of CIS and eugenol (Fathy *et al*. 2019) or wedelolactone (Sarwar *et al*. 2020). More recently, it was shown that ginsenoside combined with CIS inhibited the proliferation and migration of HeLa cells (Li *et al*. 2023).

Among the natural compounds investigated, particular attention has been given to gallic acid (GA) and its derivative, methyl gallate (MG). These compounds are commonly present in plants and herbs and have been scientifically reported to possess strong antioxidant properties (Badhani *et al*. 2015). In addition to displaying a wide range of biological activities, including antifungal and antimicrobial effects (Karamać *et al*. 2005), as well as gastroprotective, cardioprotective, anti-inflammatory and neuroprotective properties (Kahkeshani *et al*. 2019), GA and MG have shown significant antiproliferative effects against several cell lines, including HeLa cells (Mamat *et al*. 2021). Given their demonstrated anticancer potential, GA and MG warrant further investigation in the context of apoptosis, a key mechanism by which many chemotherapeutic agents—including CIS—exert their effects.

Apoptosis is a physiological process of programmed cell death that occurs when cells are no longer functional within the organism. First described by Ker *et al*. in the 1970s, apoptosis refers to cell death triggered by specific stimuli (Wong 2011). It is essential for maintaining normal cellular homeostasis, as it removes unwanted cells during development and eliminates those with DNA damage. Dysregulation of this process has been linked to cancer, atherosclerosis, respiratory disorders, chronic inflammatory disease and neurodegenerative conditions such as Parkinson’s and Alzheimer’s diseases (Redza-Dutordoir & Averill-Bates 2016).

The aetiology of cancer and several other health issues has been associated with increased production of reactive oxygen species (ROS) (Dröge 2002). The capacity of antioxidants to scavenge free radicals can counteract oxidative damage in cancer. Moreover, natural and dietary antioxidants have been shown to exhibit anticancer activity in certain types of cancer (Wang *et al*. 2016a; Owen *et al*. 2000; Widowati *et al*. 2013). Apoptosis in cancer cells may also be activated via repression of ROS signalling pathways mediated by targeted superoxide dismutase 1 (SOD1) inhibition (Li *et al*. 2019).

Considering these observations, the present study examined the possible synergistic interaction of two phenolic compounds, GA and MG, in conjunction with CIS, and further evaluated their effects on apoptotic protein production, antioxidant gene expression and cell migration in HeLa cells.

## MATERIALS AND METHODS

### Cell Treatment for Apoptotic, Antioxidant and Migration Assays

The concentrations for CIS, GA and MG alone, as well as combinations CIS–GA and CIS–MG, were adopted as suggested by Mamat *et al*. (2020). These optimal combinations were used to determine apoptotic protein production, antioxidant gene expression and to examine cell migration. The concentrations used are listed in Table 1.

These concentrations were subsequently applied in the following assays.

### Determination of Apoptotic Protein Expression by Western Blot

#### Preparation of protein lysate

Treated and untreated cells grown in 75 cm^2^ flasks were harvested after 72 h of incubation. The cells were washed twice with phosphate-buffered saline (PBS) and trypsinised, then collected in 15 mL tubes and centrifuged at 1,500 rpm for 5 min. After discarding the supernatant, 100 μL of radioimmunoprecipitation assay lysis buffer was added to lyse the cell pellet, followed by overnight incubation. The cell lysate was then centrifuged at 1,500 rpm for 5 min, and the supernatant was collected into microcentrifuge tubes. Protein concentration of the lysate was measured using a NanoDrop UV–visible spectrophotometer. Protein samples used for sodium dodecyl sulfate–polyacrylamide gel electrophoresis (SDS–PAGE) were mixed with Laemmli buffer at a 1:1 ratio and heated at 95°C for 5 min.

#### Electro transfer and western blot detection

Following separation by SDS–PAGE, proteins were transferred onto a polyvinylidene membrane using a semi-dry transblot at 15 V for 2 h. The gel was then assembled using the sandwich method between two blotting papers pre-soaked in transfer buffer in the semi-dry transblotter. After transfer, the membrane was blocked in 1% blocking solution for 1 h at room temperature. It was then incubated overnight at 4°C with minimal agitation in primary antibodies diluted in blocking buffer (β-actin, 1:1000; Bax, 1:1000; Bcl-2, 1:500; caspase-9, 1:500; caspase-3, 1:500; and p53, 1:500). The following day, the membrane was washed three times with washing buffer to remove unbound primary antibodies, then incubated with horseradish peroxidase–conjugated secondary antibody (IgG HRP, 1:1000) for 2 h at room temperature. The membrane was again washed three times with a washing buffer. Protein detection was carried out using a chemiluminescent substrate, and signals were visualised using a Fusion X chemiluminescence detection system. The integrated density value (IDV) of target protein expression relative to the control protein (β-actin) was quantified using ImageJ software. The mean relative intensity of protein expression was calculated using the following formula:


$$\text{Mean relative intensity of protein expression}=\frac{\text{IDV of targeted protein}}{\text{IDV of conrol protein}}$$

In addition to analysing apoptotic protein levels, we further examined the effect of the treatments on antioxidant-related gene expression using real-time polymerase chain reaction (PCR).

### Detection of Enzymatic Antioxidants Gene Expression by Real-Time PCR

#### Sample collection and cDNA synthesis

HeLa cells were treated with the single and combination compounds and incubated for 72 h. The spent culture medium was discarded, and the cells were rinsed twice with PBS. Cells were trypsinised with 0.25% trypsin and centrifuged at 1,500 rpm for 5 min. The resulting cell pellets were resuspended in PBS and processed for RNA extraction. Total RNA was extracted using the RNeasy Plus Mini Kit (Qiagen) according to the manufacturer’s instructions. Complementary DNA (cDNA) was synthesised from RNA using the QuantiTect Reverse Transcription Kit (Qiagen). RNA samples were immediately placed on ice after treatment with the gDNA Wipeout Buffer for 2 min at 42°C to eliminate contaminating genomic DNA. Subsequently, a master mix containing QuantiScript Reverse Transcriptase, QuantiScript RT Buffer and RT Primer Mix was used for reverse transcription. The reaction mixture was incubated at 42°C for 15 min, followed by heating at 95°C for 3 min to inactivate the QuantiScript Reverse Transcriptase.

#### Real-time PCR

All cDNA samples were amplified using an ABI StepOnePlus real-time PCR system to quantify the expression of antioxidant enzyme-related genes. Real-time PCR was performed with the QuantiFast SYBR Green PCR Kit (Qiagen). The PCR programme consisted of an initial heat activation step at 95°C for 5 min, followed by a denaturation step at 95°C for 10 sec and a combined annealing/extension step at 60°C for 30 sec. Primers used in this study were designed according to Valdameri *et al*. (2011). The forward and reverse primer sequences are shown in Table 2. The Ct values were extracted, and preliminary data were analysed using QuantStudio 5 Software. The mean relative gene expression was determined using the 2-Δ ΔCt method. Glutaraldehyde-3-phosphate dehydrogenase (GAPDH) served as the housekeeping gene and endogenous control to normalise expression levels using the comparative Ct method.

To complement these molecular assays, a wound closure assay was conducted to determine whether the treatments influenced the functional behaviour of HeLa cells, specifically their migratory capacity.

### Cell Migration Assay

Cell migration was assessed using a wound closure assay as described by Justus *et al*. (2014). Briefly, 2 × 10^5^ HeLa cells were seeded into each well of a 6-well plate. After 24 h of incubation, and once 100% confluence had been achieved, uniform vertical wounds were created in the cell monolayer using a scar scratcher. The medium and cell debris were aspirated, and the wells were rinsed with PBS. Cells were then treated with either single compounds or combinations, with untreated cells serving as controls. Images were taken at 0 h (initial) and after 24 h of incubation using an inverted microscope (Olympus, Tokyo, Japan). ImageJ software was used to measure the distance of wound closure in each well. The rate of migration was expressed as the percentage of scratch closure and calculated using the following formula:


$$\text{Percentage of scratch closure}=\frac{\left(\text{space at }0\;\text{h}-\text{space at }24\;\text{h}\right)}{\text{space at }0\;\text{h}}\times 100$$

The percentage of scratch closure was calculated for each treatment group, and these data were subsequently subjected to statistical analysis as described as follows.

### Statistical Analysis

Statistical analysis was performed using GraphPad Prism statistical software (version 7). All data are presented as mean ± standard deviation (SD) from triplicate experiments. The significance of differences among three or more groups was determined using one-way analysis of variance (ANOVA), followed by Tukey’s post hoc test. A *p*-value of < 0.05 was considered statistically significant.

## RESULTS

### Expression of Bax Protein

**FIGURE 1:**
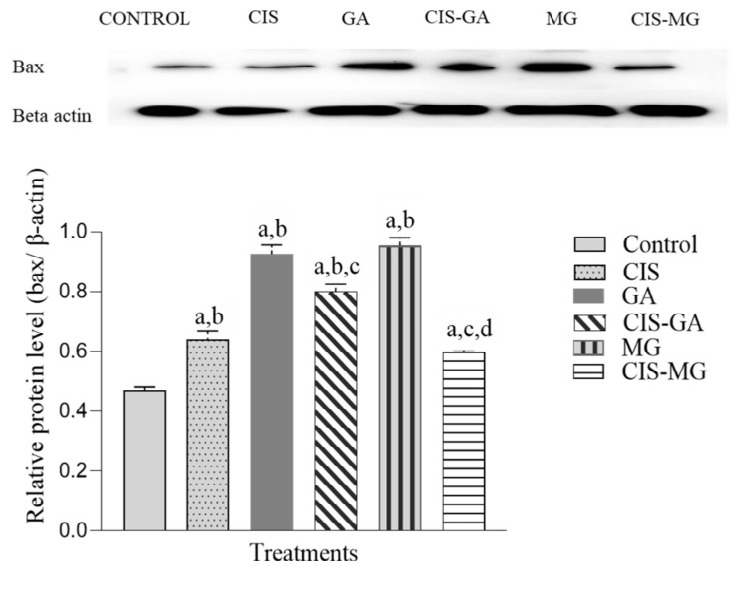
Expression of Bax in untreated and treated HeLa cells after 72 h of incubation. The graph represents the mean relative intensity obtained from IDV using ImageJ software, normalised to β-actin as an internal control. Data are presented as mean ± SD of three independent experiments. Statistical analysis was performed using one-way ANOVA followed by post hoc testing. Significance is indicated as follows: a: *p* < 0.05 vs. control; b: *p* < 0.05 vs. CIS; c: *p* < 0.05 vs. GA; d: *p* < 0.05 vs. MG.

Fig. 1 shows the relative expression level and banding pattern of Bax protein normalised to the internal control β-actin, with band detection at 23 kDa. After 72 hours of treatment, all treated groups showed significantly upregulated Bax expression compared with the untreated control group. The expression of Bax was significantly higher in cells treated with GA, CIS–GA and MG compared with CIS. However, no significant difference was observed between CIS and CIS–MG. Furthermore, Bax expression was significantly lower in the CIS–GA compared with GA, and in CIS–MG compared with MG.

### Expression of Bcl-2 Protein

**FIGURE 2:**
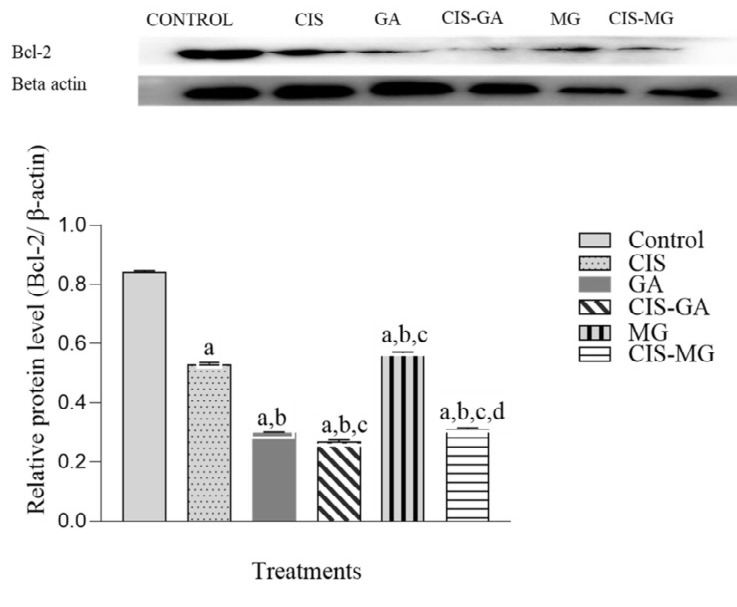
Expression of Bcl-2 in untreated and treated HeLa cells after 72 h of incubation. The graph represents the mean relative intensity obtained from IDV using ImageJ software, normalised to β-actin. Data are presented as mean ± SD of three independent experiments. Statistical analysis was performed using one-way ANOVA followed by post hoc testing. Significance is indicated as follows: a: *p* < 0.05 vs. control; b: *p* < 0.05 vs. CIS; c: *p* < 0.05 vs. GA; d: *p* < 0.05 vs. MG.

Fig. 2 shows the relative expression level and banding pattern of Bcl-2 protein normalised to the internal control β-actin, with band detection at 26 kDa. Western blot analysis revealed a marked reduction in Bcl-2 expression in all treated cells. Bcl-2 levels were significantly lower in cells treated with both single and combination treatments compared with the untreated group. In addition, significant differences in Bcl-2 expression were observed in all treated groups compared with the CIS group. Both CIS–GA and CIS–MG downregulated Bcl-2 more effectively than GA, while only CIS–MG showed significantly greater downregulation compared with MG.

### Expression of Caspase-3 and Caspase-9 Proteins

**FIGURE 3:**
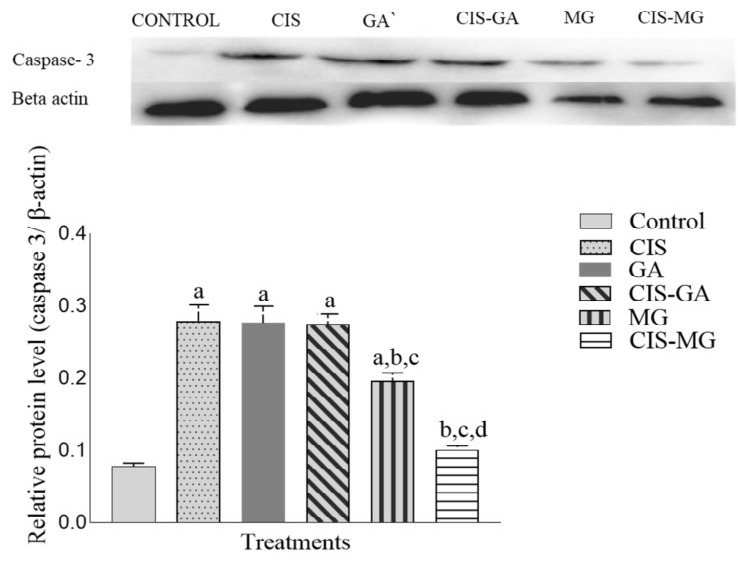
Expression of caspase-3 in untreated and treated HeLa cells after 72 h of incubation. The graph represents the mean relative intensity obtained from IDV using ImageJ software, normalised to β-actin. Data are presented as mean ± SD of three independent experiments. Statistical analysis was performed using one-way ANOVA followed by post hoc testing. Significance is indicated as follows: a: *p* < 0.05 vs. control; b: *p* < 0.05 vs. CIS; c: *p* < 0.05 vs. GA; d: *p* < 0.05 vs. MG.

All treated groups, except CIS–MG, showed significant upregulation of caspase-3 protein expression compared with the untreated group (Fig. 3). When compared with CIS, both GA and CIS–GA showed no significant differences in caspase-3 expression. No significant difference was also observed in caspase-3 expression between the GA and CIS–GA groups. However, compared with MG, caspase-3 expression in CIS–MG was significantly lower.

**FIGURE 4:**
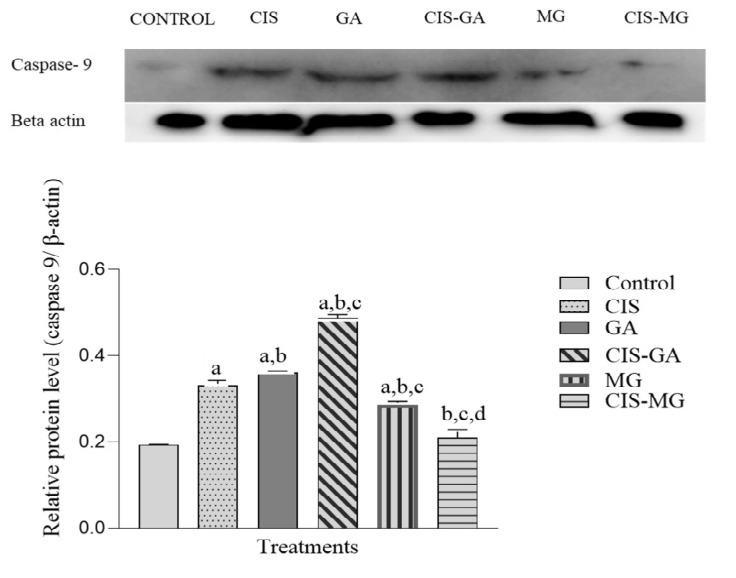
Expression of caspase-9 in untreated and treated HeLa cells after 72 h of incubation. The graph represents the mean relative intensity obtained from IDV using ImageJ software, normalised to β-actin. Data are presented as mean ± SD of three independent experiments. Statistical analysis was performed using one-way ANOVA followed by post hoc testing. Significance is indicated as follows: a: *p* < 0.05 vs. control; b: *p* < 0.05 vs. CIS; c: *p* < 0.05 vs. GA; d: *p* < 0.05 vs. MG.

To further confirm apoptotic induction, we also examined caspase-9 expression, an upstream activator of caspase-3. The expression of caspase-9 protein followed a similar trend to caspase-3 in all treated groups (Fig. 4). Marked upregulation of caspase-9 expression was observed in all treated groups except CIS–MG, with significant differences compared with the control group. When compared with CIS, caspase-9 expression was significantly higher in GA and CIS–GA, whereas it was significantly lower in MG and CIS–MG. In addition, compared with GA, CIS–GA showed significantly higher caspase-9 expression.

### Expression of p53 Protein

**FIGURE 5:**
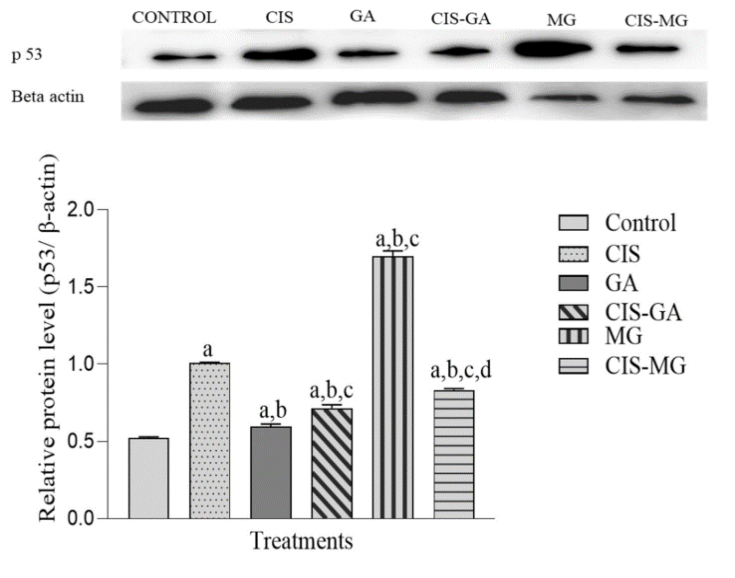
Expression of p53 in untreated and treated HeLa cells after 72 h of incubation. The graph represents the mean relative intensity obtained from IDV using ImageJ software, normalised to β-actin. Data are presented as mean ± SD of three independent experiments. Statistical analysis was performed using one-way ANOVA followed by post hoc testing. Significance is indicated as follows: a: *p* < 0.05 vs. control; b: *p* < 0.05 vs. CIS; c: *p* < 0.05 vs. GA; d: *p* < 0.05 vs. MG.

Fig. 5 shows the relative expression level and banding pattern of p53 protein normalised to the internal control β-actin, with band detection at 53 kDa. All treated groups significantly upregulated p53 expression compared with the untreated control group, with the highest expression observed in the MG group. However, when compared with CIS, p53 expression was significantly higher in the MG group and lower in cells treated with GA, CIS–GA and CIS–MG. In addition, CIS–GA showed a significantly higher p53 expression compared with GA, while CIS–MG showed a significantly lower expression compared with MG.

### Antioxidant Gene Expression Detection

**FIGURE 6:**
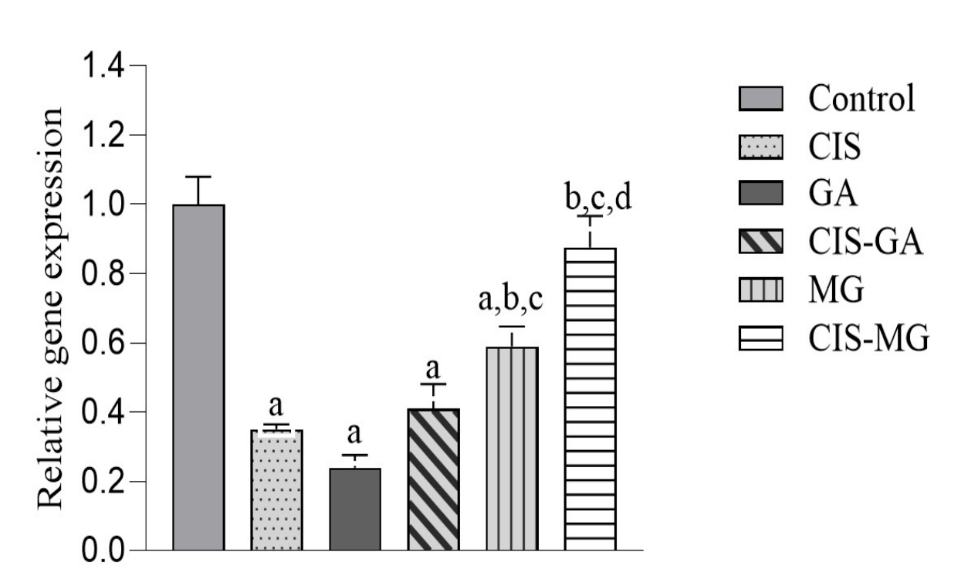
Expression of *hCAT* relative to GAPDH. All data are presented as mean ± SD of three independent experiments. Significance is indicated as follows: a: *p* < 0.05 vs. control; b: *p* < 0.05 vs. CIS; c: *p* < 0.05 vs. GA; d: *p* < 0.05 vs. MG.

Fig. 6 shows the transcriptional level of *hCAT* in treated and untreated cells. The CIS, GA, CIS–GA and MG groups significantly downregulated *hCAT* expression compared with the untreated control group. However, no significant difference was observed between CIS–MG and the control group. When compared with the CIS group, *hCAT* expression was significantly higher in the MG and CIS–MG groups. There was no significant difference between GA and CIS–GA; however, CIS–MG showed a significantly higher expression of *hCAT* compared with MG.

**FIGURE 7:**
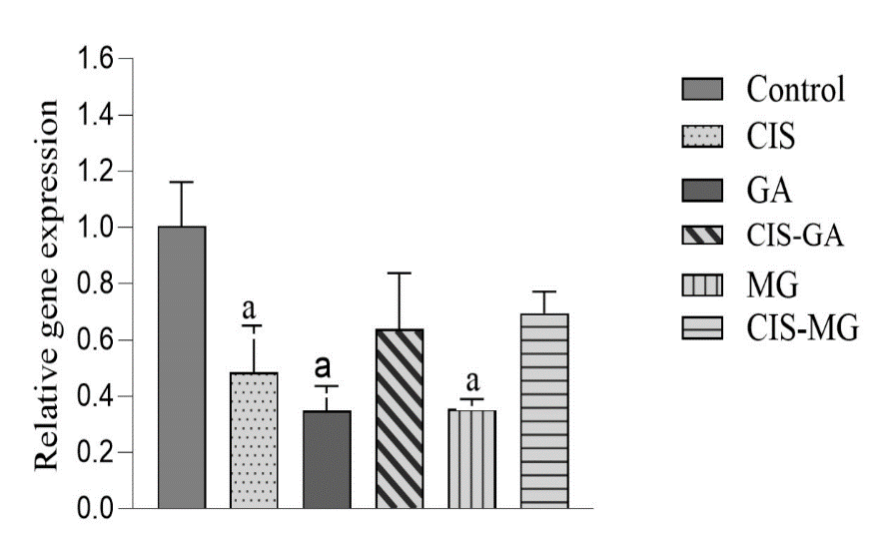
Expression of *SOD1* relative to *GAPDH*. All data are presented as mean ± SD of three independent experiments. Significance is indicated as follows: a= *p* < 0.05 vs. control.

In addition to *hCAT*, the expression of *SOD1* was also assessed. Among the treatment groups, CIS, GA and MG showed statistically significant downregulation of *SOD1* expression (*p* < 0.05) compared with the untreated control group (Fig. 7). No significant differences were observed among the treatment groups.

## Migration-Inhibitory Effect

**FIGURE 8:**
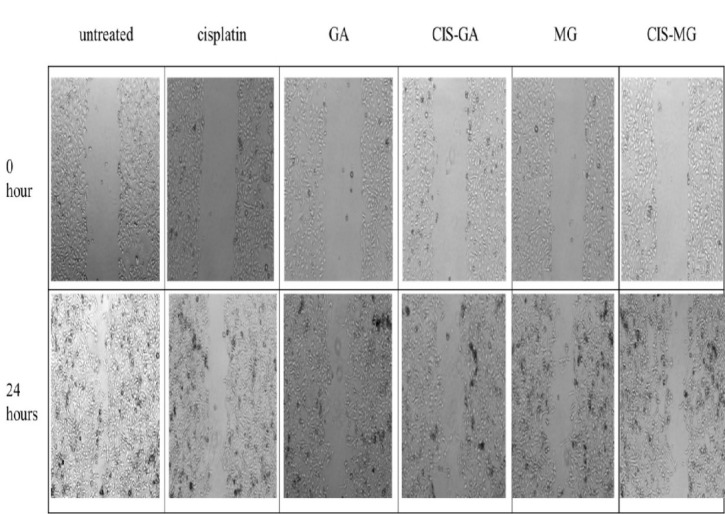
Effect of single and combination treatments of GA and MG with CIS on HeLa cell migration. Wound closure was photographed at 0 h and 24 h.

**FIGURE 9:**
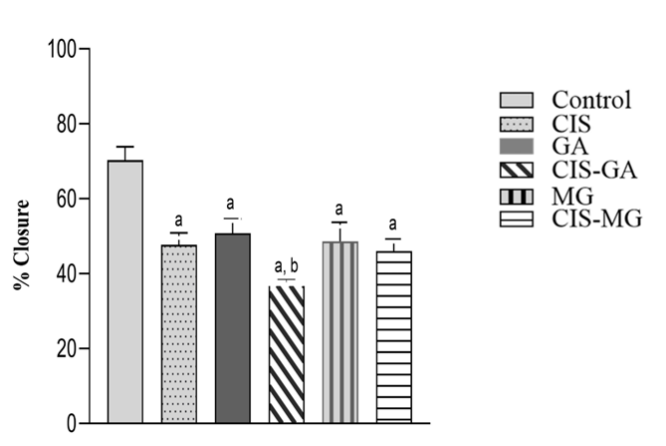
Relative wound closure of HeLa cells after 24 h of treatment. CIS–GA treatment showed the lowest wound closure. Results are presented as mean ± SD from three independent experiments. Significance is indicated as follows: a: *p* < 0.05 vs. control; b: *p* < 0.05 vs. GA.

Beyond apoptosis and antioxidant responses, we evaluated the functional effect of these treatments on cell migration. Cell migration was assessed for 24 hours post-treatment. Fig. 8 shows wound closure of the cells at 0 h and 24 h after treatment. The percentage of wound closure in cells treated with CIS (47.67 ± 3.23), GA (50.79 ± 3.83), CIS–GA (37.24 ± 1.24), MG (48.65 ± 5.0) and CIS–MG (46.06 ± 3.12) was significantly lower than in the control group (70.31 ± 3.57), as illustrated in Fig. 9. Notably, cells treated with CIS–GA showed a significantly lower percentage of wound closure compared with those treated with GA.

## DISCUSSION

Our results demonstrated that CIS, GA and MG treatments significantly modulated apoptotic protein expression in HeLa cells. To contextualise these findings, it is useful to first revisit the role of apoptosis in cancer treatment. Apoptosis is a key process in cancer treatment and can be triggered through extrinsic and intrinsic pathways regulated by death receptors and mitochondria. The extrinsic pathway involves the interaction of death ligands with death receptors, including tumour necrosis factor, Fas ligand and tumour necrosis factor-related apoptosis-inducing ligand (Obeng 2021). In contrast, the intrinsic pathway of apoptosis is triggered by p53 activation and DNA damage, initiating a series of events that ultimately result in mitochondrial outer membrane permeabilisation (Al-Aamri *et al*. 2021). Both pathways activate caspase cascades, leading to a range of morphological and biochemical alterations.

Targeting apoptosis as a mechanism for cell death has proven effective in treating different types of cancer cells and is considered one of the most successful non-surgical treatment modalities. A wide range of anticancer drugs currently used in clinical practice target both the intrinsic and extrinsic pathways. These therapeutic strategies are designed to stimulate pro-apoptotic proteins and inhibit anti-apoptotic proteins (Pfeffer & Singh 2018). As the combination of CIS–GA has been shown to induce biochemical and morphological characteristics of apoptosis in HeLa cells (Mamat *et al*. 2021), the underlying mechanism of apoptosis must be further clarified, particularly with respect to intrinsic pathways.

In the present study, we observed upregulated expression of p53 and Bax, and downregulated Bcl-2, across both single and combination treatments. Caspase-9 and caspase-3 were also upregulated, except in the CIS–MG group, where no increase was observed. These findings support the involvement of intrinsic apoptosis mechanisms induced by GA, CIS–GA and MG. The effects of GA and MG observed here are consistent with earlier studies, which reported apoptosis induction in HeLa cells mediated by upregulation of Bax, p53 and caspase-9, as well as downregulation of Bcl-2 (Abdullah *et al*. 2023). Moreover, several studies have demonstrated the pro-apoptotic effects of GA and MG in other cell lines, mediated through downregulation of Bcl-2 and upregulation of Bax and caspase-9 (Chaudhuri *et al*. 2015; Kamatham *et al*. 2015; Sun *et al*. 2016).

A study conducted by Aborehab and Osama (2019) reported that the combination of GA and paclitaxel exerted pro-apoptosis effects on HeLa cells by significantly increasing the expressions of p53 and caspase-3. Similarly, GA in combination with CIS was found to induce apoptosis through the upregulation of p53 and Bax in H446 small cell lung cancer cells (Wang *et al*. 2016b). In addition, GA was found to enhance the effect of CIS on A549 non-small cell lung cancer cells by inducing apoptosis via Bax upregulation and Bcl-2 downregulation (Zhang *et al*. 2019).

In contrast, the present study showed that CIS–MG failed to upregulate caspase-3 and caspase-9 expression. Moreover, the expression of p53, Bax and caspase-3 in cells treated with CIS–GA and CIS–MG was lower than in cells treated with GA and MG alone, suggesting that the combinations failed to produce synergistic effects. The cell death observed in these groups (CIS-GA and CIS-MG) may therefore be mediated, at least in part, by alternative mechanisms. For instance, GA has been reported to induce multiple cell death pathways simultaneously, including ferroptosis (characterised by lipid peroxidation), necroptosis (characterised by loss of plasma membrane integrity) and apoptosis (Tang & Cheung 2019). Another study demonstrated that GA combined with low-level laser irradiation, which produces higher levels of ROS compared to GA alone, resulted in increased cell death in MDA-MB-231 breast cancer cells and A375 melanoma cells via both apoptosis and ferroptosis (Khorsandi *et al*. 2020). Thus, the elevated ROS generation in CIS–GA and CIS–MG treatments may have triggered the ferroptosis pathway in HeLa cells. Given this, we next considered ROS and redox-regulated pathways in the context of our findings.

Besides apoptosis, ROS also play an important role in determining the effectiveness of cancer therapies. Acting as a secondary messenger, ROS are crucial for cancer cell growth and progression (Liou & Storz 2010). At moderate levels, ROS are essential for cell signalling and maintaining homeostasis. However, excessive ROS can damage proteins, lipids and DNA. An imbalance between ROS and antioxidant defences leads to oxidative stress, which underlies many pathological processes, including carcinogenesis (Wang *et al*. 2016b).

ROS can also promote oncogenic signalling; however, excessive accumulation is cytotoxic to cancer cells, inducing cell-cycle arrest, senescence and cell death signalling (Glasauer & Chandel 2014). Consequently, high ROS concentrations cause DNA damage in cancer cells, leading to apoptosis and tumour necrosis (Jia *et al*. 2020). Mamat *et al*. (2021) reported elevated ROS production in HeLa cells treated with the CIS–GA combination. Moreover, CIS–GA induced higher ROS levels than either treatment alone. The study also highlighted a significant increase in ROS in GA-treated HeLa cells after 72 h of treatment, consistent with other previous studies.

Mechanistically, GA itself can modulate cellular redox status, which helps contextualise its effects in our system. Apoptosis induced by GA has been linked to oxidative stress arising from several sources, including ROS, mitochondrial dysfunction and increased intracellular Ca^2+^. Interestingly, although GA is recognised as a potent antioxidant compound, it can also exert pro-oxidant effects. Its antioxidant and pro-oxidant behaviour depends on the presence of iron or hydrogen peroxide (H_2_O_2_) in the cellular environment (Park & Kim 2012). Substantial evidence has reported the pro-oxidant effects of GA in different cell lines. For example, intracellular ROS production induced by GA has been shown to trigger apoptosis in HL-60 RG human promyelocytic leukaemia cells (Inoue *et al*. 2000). Furthermore, GA isolated from *T. nigrovenulosa* extract increased intracellular ROS in HT1080 fibrosarcoma cells in a dose-dependent manner. The marked reduction of cell viability in treated cells compared with control cells suggested that at high concentrations, GA tends to exhibit stronger pro-oxidant rather than antioxidant activity (Nguyen *et al*. 2016).

Previous studies reported that combinations of GA with CIS enhance intracellular ROS production compared to untreated and single-treatment groups. This suggests that elevated ROS may be the driver of increased apoptosis in combination-treated cells, accompanied by reductions in SOD and catalase activity (Mamat *et al*. 2021). However, the current findings contradict the enzymatic antioxidant activity results of CIS–GA and CIS–MG, in which the expression of antioxidant gene (*hCAT)* was downregulated, while no changes in *SOD1* level. The decreased antioxidant enzyme activity and gene expression may be attributed to the pro-oxidant effects of CIS, GA and MG, which elevate ROS levels. However, relative to the corresponding single-agent treatments, the CIS–GA and CIS–MG combinations did not further reduce *SOD1* and *hCAT* expression. This suggests that the interaction of GA and MG with CIS in combination treatments may exert a protective influence. Overall, these results demonstrate that both single and combination treatments reduced *hCAT* expression in treated HeLa cells after 72 h of incubation. This reduction suggests that elevated ROS levels induced by these treatment compounds suppressed catalase activity, thereby promoting apoptotic cell death.

Beyond apoptotic and redox changes, we evaluated whether these treatments affected a key functional phenotype: cell migration. Cell migration plays a crucial role in cancer metastasis, making inhibition of migration an important strategy to slow its spread (Chatterjee *et al*. 2018). In this study, single treatments of GA and MG, as well as combinations of CIS–GA and CIS–MG, exhibited anti-migratory effects in HeLa cells. These findings are consistent with published work. For instance, GA inhibited HeLa cell migration after 24 h of incubation (Zhao & Hu 2013). Furthermore, GA suppressed the migration of other cell lines, including A375.S2 melanoma cells (Lo *et al*. 2011), SCC-4 human oral cancer cells (Kuo *et al*. 2014), PC-3 human prostate cancer cells (Liu *et al*. 2011) and SW1353 human chondrosarcoma cells (Liang *et al*. 2014). Similarly, MG demonstrated migration- and invasion-inhibitory effects in hepatocellular carcinoma cells (Liang *et al*. 2022).

## CONCLUSION

In this study, the combination therapy of CIS with GA and MG has been proven to induce apoptosis and reduce wound closure *in vitro* (migration proxy) in HeLa cells. The apoptotic proteins, including Bax, p53, caspase-3 and caspase-9, were significantly upregulated in the treated cells. In addition, GA and MG appeared to enhance apoptosis through their pro-apoptotic and pro-oxidant activities. Consistently, the antioxidant genes *SOD1* and *hCAT* were downregulated, indicating that targeted *SOD1* inhibition activates pathways leading to cell cycle arrest while suppressing signalling pathways that promote uncontrolled proliferation. This specific inhibition demonstrates the role of *SOD1* as a regulatory hub within the ROS signalling network, which integrates both pro-survival and pro-apoptotic signals in cancer cells.

## Figures and Tables

**TABLE 1:** Concentrations of cisplatin (CIS), gallic acid (GA) and methyl gallate (MG) used in single and combination treatments on HeLa cells.

Compound/Combination	Concentration
CIS (IC_50_)	8.04 μg/mL
GA (IC_50_)	13.44 μg/mL
MG (IC_50_)	16.55 μg/mL
**CIS–GA Combination**	
CIS	0.51 μg/mL
GA (IC_50_)	13.44 μg/mL
**CIS–MG Combination**	
CIS	0.51 μg/mL
MG (IC_50_)	16.55 μg/mL

**TABLE 2:** List of primers.

Gene	Primer	Sequence
*SOD1*	Forward	5′-GAA GGT GTG GGG AAG CAT TA-3′
	Reverse	5′-CCC ACC GTG TTT TCT GGA TA-3′
*hCAT*	Forward	5′-GATAGCCTTCGACCCAAGCA-3′
	Reverse	5′-ATGGCGGTGAGTGTCAGGAT-3′
*GAPDH*	Forward	5′-ACCCACTCCTCCACCTTTGA-3′
	Reverse	5′-CTGTTGCTGTAGCCAAATTCGT-3′
